# Fragile X targeted pharmacotherapy: lessons learned and future directions

**DOI:** 10.1186/s11689-017-9186-9

**Published:** 2017-06-12

**Authors:** Craig A. Erickson, Matthew H. Davenport, Tori L. Schaefer, Logan K. Wink, Ernest V. Pedapati, John A. Sweeney, Sarah E. Fitzpatrick, W. Ted Brown, Dejan Budimirovic, Randi J. Hagerman, David Hessl, Walter E. Kaufmann, Elizabeth Berry-Kravis

**Affiliations:** 1 Division of Child and Adolescent Psychiatry (MLC 4002), Cincinnati Children’s Hospital Medical Center, 3333 Burnet Ave., Cincinnati, OH 45229-3039 USA; 2 Department of Psychiatry, College of Medicine, University of Cincinnati, Cincinnati, OH USA; 3 Department of Biomedical Engineering, College of Engineering and Applied Science, University of Cincinnati, Cincinnati, OH USA; 4 Medical Investigation of Neurodevelopmental Disorders (MIND) Institute, Davis Medical Center, University of California, Sacramento, CA USA; 5 Department of Pediatrics, Davis Medical Center, University of California, Sacramento, California USA; 6 Department of Psychiatry and Behavioral Sciences, Davis Medical Center, University of California, Sacramento, California USA; 7 Greenwood Genetic Center, Greenwood, SC USA; 8 Boston Children’s Hospital, Boston, Massachusetts USA; 9 Departments of Pediatrics, Neurological Sciences, Biochemistry, Rush University Medical Center, Chicago, Illinois USA; 10 Institute for Basic Research in Developmental Disabilities, New York, NY USA; 11 Clinical Research Center, Clinical Trials Unit, Fragile X Clinic, Kennedy Krieger Institute, The Johns Hopkins Medical Institutions, Baltimore, MD USA; 12 Departments of Psychiatry & Behavioral Sciences, Child Psychiatry, The Johns Hopkins Medical Institutions, Baltimore, MD USA

**Keywords:** Fragile X syndrome, Translational treatment, Targeted treatments, Drug development, Genetic disorder

## Abstract

Our understanding of fragile X syndrome (FXS) pathophysiology continues to improve and numerous potential drug targets have been identified. Yet, current prescribing practices are only symptom-based in order to manage difficult behaviors, as no drug to date is approved for the treatment of FXS. Drugs impacting a diversity of targets in the brain have been studied in recent FXS-specific clinical trials. While many drugs have focused on regulation of enhanced glutamatergic or deficient GABAergic neurotransmission, compounds studied have not been limited to these mechanisms. As a single-gene disorder, it was thought that FXS would have consistent drug targets that could be modulated with pharmacotherapy and lead to significant improvement. Unfortunately, despite promising results in FXS animal models, translational drug treatment development in FXS has largely failed. Future success in this field will depend on learning from past challenges to improve clinical trial design, choose appropriate outcome measures and age range choices, and find readily modulated drug targets. Even with many negative placebo-controlled study results, the field continues to move forward exploring both the new mechanistic drug approaches combined with ways to improve trial execution. This review summarizes the known phenotype and pathophysiology of FXS and past clinical trial rationale and results, and discusses current challenges facing the field and lessons from which to learn for future treatment development efforts.

## Background

Fragile X syndrome (FXS) is the most common single gene disorder associated with autism spectrum disorder (ASD) and most common inherited cause of developmental disability. FXS impacts 1 in 4000 males and 1 in 4000–6000 females worldwide [1–5]. FXS results from silencing of the fragile X mental retardation gene (*FMR1)* on the long arm of the X chromosome. Silencing of *FMR1* is nearly always caused by hypermethylation of a cytosine guanine guanine (CGG) trinucleotide repeat expansion (≥200 repeats is termed the “full mutation” and causes FXS) in the 5′ untranslated region (UTR) of the *FMR1* gene [6, 7]. *FMR1* inactivation results in absent or deficient production of fragile X mental retardation protein (FMRP). In all cases, full mutation FXS results from maternal transmission, in which a mother transmits her full mutation allele or her premutation (carrier) allele (55–200 CGG repeats; typical population has fewer than 45 repeats), which undergoes CGG repeat expansion when it transmits to the next generation. As an X-linked disorder, FXS universally impacts affected males, while its presentation is variable in females due to random X inactivation patterns. In FXS, excessive and poorly regulated protein synthesis is pathogenic, which then manifests in myriad ways [8]. Developmental disability, most commonly in the moderate to severe range of cognitive impairment, is universal in males. Common physical and medical features in FXS include increased risk for chronic otitis media, esotropia, hyperextensible joints, high arched palate, low muscle tone, seizures, and macroorchidism with puberty [9, 10]. The neurobehavioral presentation of FXS includes risk for sleep disturbance, aggression, attention deficit hyperactivity disorder (ADHD) symptoms, significant anxiety, sensory hypersensitivities, self-injury, and physical aggression [4, 11, 12]. There is a significant overlap between FXS and ASD, with up to 2 in 3 males with FXS having features consistent with the broader ASD phenotype [3, 5, 13, 14].

FMRP is widely expressed in humans. In the human brain, FMRP is expressed in mature astrocytes and in the dendrites, spines, and soma of mature neurons [15–19]. FMRP is involved in translational repression and thought to selectively bind to about 4% of all mRNAs translated in the brain [17–20], but the impact of deficient FMRP is complex, including an expected increase in translation of many RNA targets. However, protein expression of other mRNA targets of FMRP may be unchanged or even reduced in FXS, thus pointing to poorly understood compensatory or other regulatory mechanisms [21].

The loss of FMRP results in a number of brain effects at macroscopic, microscopic, and molecular levels. Neuroimaging abnormalities noted in youth with FXS include larger temporal lobe white matter, cerebellar gray matter, and caudate nucleus with smaller amygdala compared to controls [22]. At a microscopic level, dendritic abnormalities associated with deficient FMRP include increased spine density with longer, spindly, and immature morphology reported in postmortem human and *Fmr1* knockout (KO) mouse brain tissue [23–25]. Molecular and dendritic abnormalities may result in functional brain deficits, including disruption of synaptic plasticity with enhanced long-term depression (LTD) [26–29] and brain region-specific long-term potentiation (LTP) deficits [30–33]. Not surprisingly, given the large number of proteins for which production is altered by FMRP deficiency, many molecular signaling cascades involved in synaptic plasticity, learning, and memory are known to function abnormally in the *Fmr1* KO mouse and in human cells. Some of these likely dysregulated molecular systems include phosphoinositide 3-kinase (PI3K) [34–36], extracellular signal-related kinase (ERK1/2) [37–39], matrix metalloproteinase 9 (MMP-9) [40, 41], endocannabinoid [42–45], brain-derived neurotrophic factor (BDNF) [46, 47], and mammalian target of rapamycin (mTOR) [48–50]. The broad array of potential molecular targets for pharmacotherapy in FXS is a testament to the broad impact of deficient FMRP, and thus the potential challenge of targeting multiple aspects of molecular dysregulation simultaneously [42–45].

As a single gene disorder with increasing efforts to define and then address the neurobiological underpinning of the disorder, FXS has been the subject of a recent wave of targeted treatment development efforts. Despite significant hopes for translational treatment success, to date no drug has met approval for use specifically in FXS. Given this, the pharmacotherapy of FXS in clinic continues to be limited to symptomatic treatments of comorbid abnormal behaviors, employing medications such as selective serotonin reuptake inhibitors (SSRIs), stimulants, and second-generation (atypical) antipsychotics [51]. We will focus on efforts to develop FXS-specific pharmacotherapy, including the translational basis for various treatment hypotheses, focused on results of human studies. Finally, we will look in detail at potential reasons for study failure providing future directions for consideration to address such challenges.

## Modulation of glutamate and GABA neurotransmission

Many of the recent targeted clinical trials in FXS have addressed a potential excitatory/inhibitory neurotransmission imbalance associated with the disorder (Table 1). In the FXS brain, there is believed to be an excess of excitatory, glutamatergic signaling coupled with deficiencies in inhibitory, γ-Aminobutyric acid (GABA)-ergic signaling [52]. Several recent human trials in FXS have focused on reducing excitatory glutamatergic neurotransmission. Specific glutamatergic effects probed in humans with FXS have included antagonism of group I metabotropic glutamate receptors (mGluRs), particularly mGluR5 (ClinicalTrials.gov Identifiers: NCT01253629, NCT01357239, NCT01517698, NCT01015430, NCT01750957), the N-methyl-D-aspartate (NMDA) receptor, stimulation of GABA(B) receptors (NCT01325220, NCT00788073, NCT01282268), and modulation of the α-amino-3-hydroxy-5-methyl-4-isoxazolepropionic acid glutamate receptor (AMPAR; NCT00054730).

**Table 1:** Clinical trials to date in fragile X syndrome by drug and study type

Drug	Mechanism	Open Label/Chart Review	Phase II	Phase III
Acamprosate	GABA-R agonist	C[98]; C[99]	O	
Arbaclofen	GABA_B_-R agonist		C[102]	P
Basimglurant	mGlur5 antagonist		P	
CX516	AMPAR (+) modulator		C[85]	
Donepezil	Anticholinergic	C[132]	C[134]	
Fenobam	mGlur5 antagonist	C[73]		
Ganaxolone	GABA_A_R agonist		O	
Lithium	GSK3 inhibitor	C[114]		
Lovastatin	ERK inhibitor	C[125]		
Mavoglurant^a^	mGlur5 antagonist		C[75], C[76, 106]	D
Memantine	NMDAR antagonist	C[84]		
Metadoxine ER	GABA agonist		P	
Minocycline	MMP9 inhibitor	C[117]	C[117]	
Oxytocin	OXTR agonist		C[143]	
Riluzole	Glutamate agonist	C[93]		
R04917523	mGlurR5 antagonist		D	
Trofinetide^b^	IGF-1 mimic		P	

Key: *C* completed and published, *P* completed and pending publication, *O* ongoing clinical trial, www.clinicaltrials.gov, *D* discontinued and unpublished

^a^AFQ056

^b^NNZ-2566

The mGluR theory of FXS pathophysiology has driven the wave of glutamatergic modulator studies in FXS. The mGluR theory postulates that in FXS, excessive signaling through mGluRs contributes to behavioral, electrophysiological, and molecular dysfunction associated with the disorder [8]. The mGluR theory brought together several key findings, including observations that FMRP represses protein translation at the synapse [53], synaptic protein synthesis can be triggered by activation of mGluRs [16], FMRP deficit leads to increased downstream effects of mGluR signaling [26, 54], and many of these downstream effects are dependent on mRNA translation at the synapse [55–58]. The mGluR theory has been extensively corroborated in FXS preclinical research using 2-methyl-6(phenylethynyl)pyridine (MPEP), a selective mGluR5 antagonist neurotoxic to humans. This work has included many studies of treatment of the *Fmr1* KO mouse with selective mGluR5 antagonists, demonstrating rescue of aberrant AMPAR expression, behavioral deficits, electrophysiological abnormalities, protein expression dysregulation, and altered dendritic spine morphology [29, 59–61]. Further corroboration of the mGluR theory has come from genetic knockdown studies in the *Fmr1* KO mice, in which reduction in mGluR5 expression normalized protein synthesis, dendritic spine deficits, and aspects of aberrant behavior [62], although a second study using the same model found very few behavioral improvements [63].

Within investigation of excitory/inhibitory imbalance, enhanced glutamatergic signaling has received the most attention in FXS, but GABAergic deficits have been increasingly recognized as contributing to the hypothesized excitatory/inhibitory imbalance. In the *Fmr1* KO mouse, deficits in GABAergic signaling occur in a variety of brain regions including the hippocampus, striatum, amygdala, and somatosensory cortex [64–67]. Most commonly, preclinical findings of GABAergic deficits have centered on reduction in GABA(A) subunit receptor expression, although GABA synthesis and release may also be affected [64]. In preclinical treatment study in the mouse and fly model of FXS, positive modulation of GABA(A) receptors can rescue some behavioral and neurophysiological alterations [68, 69]. GABA(B) activators also rescued an array of phenotypes including spine morphology, audiogenic seizures, and cellular abnormalities [70, 71]. Overall, the imbalance of glutamatergic and GABAergic signaling in FXS may represent a complex interplay of abnormalities on both sides of the excitatory/inhibitory neurotransmission balance.

Phenotypic rescue demonstrated in the *Fmr1* KO mouse, first with use of selective mGluR5 antagonists and later with other mechanistic approaches, has driven the extensive clinical trial work in the field since 2008. To date, a total of 22 such studies have been identified through search of literature and other sources; 19/22 (86%) have been registered on www.ClinicalTrials.gov. As expected from FXS neurobiology, the vast majority of the studies have targeted the core excitatory/inhibitory imbalance in the disorder primarily through either mGluR5 antagonists (mavoglurant-AFQ056, NCT01357239, NCT01253629; basimglurant-RO4917523, NCT01517698, NCT01750957) or GABA agonists (arbaclofen-GABA-B agonist, NCT01282268, NCT00788073, NCT01325220; ganaxolone-GABA-A agonist, NCT01725152). These studies represent the majority of the total (14/22, 64%) and FDA-registered (14/19, 74%) trials. Reflecting that over 2/3 of these trials were phase II, most of them have studied adults and adolescents (i.e., regulations specify that novel drugs need to be tested first in adults, particularly in vulnerable populations). Trials of three specific mGluR5 antagonists—fenobam, mavoglurant (AFQ056), and basimglurant (RO4917523)—in human FXS study have been completed. The first pilot trial involved fenobam [N-(3-chlorophenyl)-N'-(4,5-dihydro-1-methyl-4-oxo-1H-imidazole-2-yl)urea], a nonbenzodiazepine anxiolytic drug and negative allosteric modulator of mGluR5 [72]. An open-label single-dose study was conducted in 6 males and 6 females with FXS in the 18 to 30-year-old range [73]. This pilot study initially evaluated drug safety and pharmacokinetics and explored aspects of sensory gating, attention and inhibition by evaluating prepulse inhibition (PPI) before and after each single dose fenobam treatment. No significant adverse events were noted. Six out of 12 (50%) subjects met predefined response criterion of at least 20% improvement over baseline on PPI at 120 ms. Further studies in FXS were not carried out because the company that manufactured fenobam failed financially. In earlier reports in the general population, fenobam has a challenging tolerability profile, with reports of hallucinations, vertigo, paraesthesias, and insomnia with fenobam use [74].

Mavoglurant is a noncompetitive mGluR5 antagonist developed by Novartis Pharmaceuticals. Three placebo-controlled trials of mavoglurant have been completed in FXS. The first trial was a double-blind, placebo-controlled 20-day treatment period crossover trial including 30 adults with full mutation FXS [75]. While this initial study failed to show any positive drug-associated effect on primary or secondary outcomes, including the Aberrant Behavior Checklist (ABC) or Clinical Global Impressions (CGI) scales, in the whole study group, a post-hoc subset analysis of seven individuals with complete *FMR1* promoter methylation noted significant drug-associated improvement on a number of behavioral outcome measures. This post-hoc effect may have been driven by an abnormally low placebo response in the small seven-subject subset of patients with complete methylation. This finding could also could be attributable to regression to the mean with those with complete methylation with a potentially more significantly impaired phenotype. Two additional large-scale double-blind, placebo-controlled, parallel groups, fixed-dose four-arm (placebo, 25 mg BID, 50 mg BID, and 100 mg BID dosing) studies with open label extension were conducted with mavoglurant in FXS [76]. The trials included 12-week adult (age range 18–45 years) and adolescent (age range 12–17 years) placebo-controlled treatment periods, both utilizing the total score of a FXS-refactored version [77] of the Aberrant Behavior Checklist-Community (ABC-C) [78], termed ABC_FX_, as a primary outcome. Neither study met significance on the primary endpoint and the sponsor subsequently terminated the open-label extension portion of the studies and discontinued the development program of mavoglurant in FXS (ClinicalTrials.gov Identifiers: NCT01253629, NCT01357239); [76, 79].

A third selective mGluR5 antagonist, basimglurant, was evaluated in a small phase IIa placebo-controlled PK study in adults, a subsequently a larger phase IIb 3-month double-blind, placebo-controlled study in adolescents and adults (age range 14-50 years) (ClinicalTrials.gov Identifier: NCT01517698), and a small phase IIa pharmacokinetic study in youth (age range 5-13 years) with FXS (ClinicalTrials.gov Identifiers: NCT01015430, NCT01750957). Although all the trial results remain unpublished, due to lack of efficacy on a number of behavioral and other outcome measures employed in phase IIb adult/adolescent study, the Roche Group subsequently terminated its program for the development of basimglurant in FXS [80].

Mavoglurant and basimglurant trial results have made it clear that short-term selective mGluR5 antagonism is not associated with significant behavioral improvement in the age ranges studied. Several questions remain to be answered, including what impact this class of drugs may have on very young children and what the impact of prolonged treatment may be on other outcomes such as cognitive or communication metrics. The selective mGluR5 antagonist trials to date have also been limited by the employed outcome measures, with a focus on parent-reported behavioral outcomes that resulted in dramatically enhanced placebo response.

In addition to evidence of mGluR5 dysregulation in FXS, activity at the NMDA glutamate receptor may be anomalous in the disorder, although the overall directionality of dysregulation is unclear, appearing in preclinical models to depend on brain region and developmental stage [26, 81–83]. Memantine (3,5-dimethyladamantan-1-amine), a compound that acts as a NMDA noncompetitive antagonist, is the US Food and Drug Administration (FDA) approved for the management of Alzheimer’s disease. In the only published trial of memantine in humans (age range 13–22 years, *n* = 6) with FXS to date, over a mean 34.7 weeks of open-label treatment, 4 subjects showed clinical improvement as rated by the Clinical Global Impression–Improvement subscale (CGI-I), but 2 subjects had to discontinue therapy due to increased irritability with treatment [84]. No specific domains of symptom or behavioral improvement were noted and the authors cautioned about future exploration of this drug given the worsening irritability noted in one third of subjects.

AMPAR is an ionotropic glutamate receptor mediating fast synaptic transmission. Modulation of AMPAR activity downstream of mGluR signaling, was initially proposed as a method of restoring excitatory:inhibitory signaling balance in FXS [8]. Level of internalization of AMPARs, which is increased in the *Fmr1* KO mouse, may contribute to alterations in LTD and LTP in FXS since AMPAR signaling is required for maintaining synaptic plasticity. A positive allosteric modulator of AMPAR, CX516, was tested in a 4-week double-blind, placebo-controlled trial in FXS. The trial failed to find a significant treatment-associated improvement in memory, the study’s primary measure, or any secondary endpoint including measures of language skills, behavior, and global improvement. The authors hypothesized that CX516 drug effect may have been limited by potential subtherapeutic dosing based on information that became available concurrent to the trial [85]. Despite the negative study findings, the CX516 trial laid the groundwork for the use of many outcome measures in future FXS trials by providing invaluable data regarding which measures may not be as affected by floor, ceiling, or test-retest inconsistency effects.

Beyond drugs solely modulating glutamatergic neurotransmission, several agents with combined glutamate and GABA activity have been studied in FXS. Dysregulation of the ERK intracellular signaling pathway has been implicated in the pathophysiology of FXS and as such has become a potential target of treatment in the disorder. Acting downstream from several cellular receptors including mGluRs, ERK activity is required for normal synaptic plasticity and the regulation of activity dependent protein synthesis [86]. ERK activity has been demonstrated to be upregulated under baseline conditions in the *Fmr1* KO mouse and in human post-mortem tissue [28, 49, 87]. The kinetics of ERK activation is delayed in FXS, potentially because of baseline hyperactivation. Reduction of phosphorylated (activated) ERK has been linked to rescuing the audiogenic seizure phenotype [87] and the increased hippocampal protein synthesis in the *Fmr1* KO mouse [88].

Riluzole is FDA-approved for the treatment of amyotrophic lateral sclerosis (ALS) and may potentially be useful in the treatment of depression and anxiety [89, 90]. The drug is hypothesized to inhibit glutamate release [91] and potentiate post-synaptic GABA(A) receptor activity [92]. Riluzole was the subject of a small six subject 6-week open-label study in adult males with FXS [93]. Only 1 in 6 subjects (16%) showed a positive clinical response after the brief treatment (100 mg/day). Despite the lack of significant clinical effect, peripheral lymphocytic ERK activation, which is known to be delayed in blood lymphocytes in FXS human and animal models [94], was significantly corrected in all subjects following the 6-week riluzole treatment. This result, potential rescue of molecular dysregulation combined with a lack of clinical response, may be a signal that short-term trials are not allowing the time for molecular change to generate resultant positive change in the clinical phenotype of FXS. It may also be possible that ERK dysregulation is a downstream consequence of a different molecular mechanism and thus an isolated correction of ERK activity could lack significant clinical impact.

Acamprosate is FDA-approved for the maintenance of abstinence in alcohol dependence. Acamprosate is hypothesized to have combined effects on excitatory:inhibitory balance in brain, including potential potentiation of GABA(A) activity [95] and antagonism at mGluR5 [96] and NMDA glutamate receptors [97]. Acamprosate has been the subject of several open-label reports in FXS including an initial report on 3 adults with FXS who received a mean 21.3 weeks of acamprosate treatment [98]. In this study, each adult showed a positive clinical treatment response marked in part by enhancement in language and communication skills. In the first investigation of acamprosate in youth with FXS, over 10 weeks of open-label treatment (mean dose 1054 ± 422 mg/day), 9 of 12 youths were deemed treatment responders with response marked by a score of “much” or “very much improved” on the CGI-I [99]. In addition, overall group improvement was noted on the ABC-C Social Withdrawal subscale (ABC-SW), Social Responsiveness Scale (SRS), and attention deficit hyperactivity disorder rating scale (ADHD-RS). In this report, plasma BDNF was sampled at baseline and after acamprosate treatment. Acamprosate use was associated with increases in peripheral BDNF levels after 10 weeks of treatment. Additionally, plasma amyloid precursor protein (APP) and APP alpha were reduced with acamprosate use in this trial [100]. Overall, in open-label studies, acamprosate has shown promise to improve the clinical phenotype of FXS and the drug has been associated with two aspects of molecular change that may signal aspects of engagement with the underlying molecular dysregulation that characterizes the disorder. Acamprosate is currently undergoing double-blind, placebo-controlled 10-week study in 48 persons with FXS age 5–22 years (ClinicalTrials.gov Identifier: NCT01911455).

The neuroactive steroid ganaxolone (3a-hydroxy-3B-methyl analog of allopregnanolone) is a positive allosteric modulator at GABA(A) receptors. Ganaxolone has blocked audiogenic seizures in the *Fmr1* KO mouse [101]. A double-blind, placebo-controlled 6-week treatment period, crossover trial of ganaxolone was recently completed in 6–17 years old with FXS (ClinicalTrials.gov Identifier: NCT01725152), although results have not yet been published or presented. This first in FXS ganaxolone study aimed to determine the safety, tolerability, and efficacy of the drug for the treatment of anxiety and attention deficits in FXS.

Arbaclofen, the active entaniomer of racemic baclofen, is a GABA(B) agonist studied to date in FXS and in idiopathic ASD. Arbaclofen, a presynaptic GABA(B) agonist, is postulated to inhibit glutamatergic release, thus potentially limiting neuronal hyperexcitability associated with FXS. In the *Fmr1* KO mouse, arbaclofen was shown to reduce susceptibility to audiogenic seizures and normalize excessive dendritic spine density and protein synthesis [70]. In a double-blind, placebo-controlled 4-week treatment period crossover trial in 63 persons with FXS age 6–40 years, the drug was well tolerated but not associated with a positive drug effect on the study primary outcome, the ABC-C Irritability subscale (ABC-I) [102]. Other outcomes, including a parent visual analog scale (VAS) of the child’s 3 most challenging behaviors reported by caregivers and the ABC Social Avoidance (ABC-SA) subscale, which was developed specifically by re-factoring the ABC-C in the FXS population (ABC_FX_) [77] were improved on arbaclofen in the entire per intent-to-treat (ITT) treatment group. In post-hoc analyses, a more socially impaired subgroup defined by high ABC-C Social Withdrawal (ABC-SW) scores at baseline showed a positive drug-associated treatment effect on many measures including the CGI-Severity (CGI-S), CGI-I, treatment preference, VAS, and ABC_FX_ Social Avoidance subscale. Subsequent phase III studies of arbaclofen in FXS across children, adolescents, and adults did not show significant drug-associated improvement on primary outcome measures tested (Berry-Kravis et al. 2016, companion paper in this issue of the Journal). The pediatric study did show arbaclofen-associated broader measures improvement on several key secondary outcomes and a trend toward significance for arbaclofen on the primary endpoint, but the study was not adequately powered for the primary outcome measure due to early closure for financial reasons. These results combined with parallel negative phase III findings in idiopathic ASD led to the discontinuation of the development of arbaclofen by Seaside Therapeutics, when the company ceased operations.

## Targeted treatment development beyond glutamate and/or GABA modulation

Metadoxine (pyridoxol l-2-pyrrolidone-5-carboxylate) has been used to treat alcohol intoxication outside of the USA for many years. Metadoxine ER (MDX), which has been shown to increase striatal dopamine levels in murine models [103], is currently being developed by Alcobra Pharmaceuticals for use in adult and pediatric ADHD. MDX is currently in phase III development for adults with ADHD (ClinicalTrials.gov Identifiers: NCT02477748 and NCT02189772). In the *Fmr1* KO mouse, as reported by Alcobra but not available in a peer-reviewed manuscript format, MDX use was associated with improvements in attention, memory, learning, hyperactivity, and sociability, which were associated withmolecular normalization of Akt and ERK over-activity (http://www.alcobra-pharma.com/releasedetail.cfm?ReleaseID=847048). A 6-week randomized, double-blind, placebo-controlled trial of MDX enrolling 62 persons (57 completed treatment) with FXS, age 14–55 years (mean age: 24 years), was recently completed (ClinicalTrials.gov Identifier: NCT02126995) [104]. MDX use was not associated with significant improvement on the study’s primary outcome measure: the inattentive subscale of the ADHD Rating Scale 4th Edition (ADHD-RS-IV). An analysis of secondary outcomes included a positive report of MDX-associated benefit as measured by the Vineland Adaptive Behavior Scale, Second Edition (Vineland-II) Daily Living Skills Domain [76, 104, 105] and the computerized cognitive Test of Attentional Performance for Children (KiTAP) Go-NoGo subscale false reactions (*p* = 0.043). Although improvement on the Vineland-II would be an important functional outcome in FXS, future work including replication is necessary to further understand any potential utility of MDX use in persons with FXS.

Lithium is an effective mood stabilizer, FDA-approved for the treatment of bipolar disorder. The drug has combined effects including inhibition of glycogen synthase kinase-3 beta (GSK-3b). GSK-3b has been shown to be dysregulated in the *Fmr1* KO mouse [106, 107]. In this model of FXS, lithium use has been associated with improvements in hyperactivity, social preference, learning, and aberrant dendritic spine development [108]. Lithium has also been shown to rescue synaptic plasticity, protein synthesis, and aberrant GSK-3b activity in the *Fmr1* KO mouse [109–112]. Lithium has been evaluated in a 15-person open-label 2-month study in FXS [113]. In this report, lithium use was not associated with significant improvement on the ABC-I, but did show treatment-associated improvement on a number of secondary outcome measures including other ABC-C subscales, the ABC-C total score, the CGI-I, a visual analog scale (VAS) for behavior, the Repeatable Battery for the Assessment of Neuropsychological Status (RBANS) List Learning subtest assessing verbal memory, and ERK activation in lymphocytes. Adverse effects associated with lithium use in this trial included aggression, polydipsia, and enuresis. The side effect profile of lithium including risk of thyroid and renal impairment has limited further development of this compound in FXS.

Minocycline, an FDA-approved antibiotic treatment for acne in youth, is known to have inhibitory effects on MMP-9 activity. MMP-9 activity has been demonstrated to be elevated in the hippocampus of *Fmr1* KO mice [114]. In this mouse model of FXS, minocycline treatment was associated with reduced hyperactivity and improvement in dendritic spine phenotype [115]. In an initial 8-week open-label minocycline trial in 20 persons with FXS age 13 to 35 years, the drug treatment was associated with widespread improvement as captured by the CGI and ABC subscales [116]. A subsequent 12-week treatment period double-blind, placebo-controlled crossover trial in 55 subjects age 3.5 to 16 years with FXS noted drug-associated improvement on the CGI-I, but no group-wide improvement in specific behavioral domains [117]. A post-hoc analysis noted improvement on the VAS specific to anxiety and mood-related concerns. In an electrophysiology analysis of a 12-subject subgroup from the placebo-controlled minocycline trial, minocycline use was associated with improvement in habituation to auditory stimuli as shown by an event-related potentials (ERP) passive auditory oddball paradigm [118]. The authors hypothesized that this electrophysiological improvement in habituation may be related to improvements in hypersensitivity to auditory stimuli noted in humans with FXS following minocycline administration. Dziembowska et al. (2013) demonstrated that MMP-9 blood level was significantly elevated in 20 patients with FXS compared to controls; 6 of the 10 children treated with minocycline for 12 weeks showed a significant lowering of their MMP-9 levels, although their response on the CGI-I did not correlate with the degree of MMP-9 lowering. Although minocycline is recognized primarily for lowering MMP-9, it has multiple other effects including stalling translation, decreasing apoptosis, and working as an antioxidant, all of which may be helpful in FXS [119].

Lovastatin is a compound FDA-approved for the long-term management of familial hyper-cholesterolemia [120], with demonstrated effects on intracellular signaling. In cultured rat brain neuroblasts, lovastatin was shown to inhibit Ras signaling, an upstream effect that resulted in reduction in ERK activation [121] that supported previous work in fibroblasts [122]. In *Fmr1* KO mice, lovastatin was confirmed to inhibit Ras, reduce increased basal ERK activation, lower protein synthesis to wild type levels, and ameliorate FXS audiogenic seizure susceptibility [123]. Based upon the known safety profile of lovastatin and the aforementioned promising preclinical results, efficacy of lovastatin in FXS was assessed in a 16-person, open-label trial of children and adolescents. Treatment response was assessed using the ABC-C, CGI-I, and Vineland-II. Significant improvement was observed after 4 and 12 weeks of treatment, with the ABC-C, CGI, and Vineland-II scores improving from week 4 to week 12. Excessive ERK activity measured in platelets was reduced by lovastatin in this trial and correlated with behavioral improvement on the ABC-C. There was modest improvement on the CGI-I, but the open-label nature of the trial precludes any strong inferences of efficacy at this stage of development [124]. Furthermore, particular importance should be placed on lipid monitoring in future lovastatin trials since individuals with FXS were reported to have lower levels of low and high density lipoprotein and total cholesterol [125].

A synthetic analog of the naturally occurring active N-terminal tripeptide derived of the insulin-like growth factor 1 (IGF-1), known as NNZ-2566 or trofinetide has been studied in the *Fmr1* KO mouse and in humans with FXS. The drug was initially developed for traumatic brain injury, where trofinetide showed promise with improved recovery, reduction in apoptotic cell death, and reduced neuroinflamation noted in a rat model [126–128]. In the *Fmr1* KO mouse, trofinetide has been reported to rescue learning and memory deficits, normalize dendritic spine morphology, and restore normal ERK signaling [129]. Recently, a double-blind, placebo-controlled 4-week trial of NNZ-2566 in 12 to 45 year old males with FXS was completed. The study used both parent- and clinician-reported outcomes designed to address the entire FXS phenotype, including an FXS Rating Scale and FXS Domain Specific Concerns measure. A composite analysis of five measures from three domains utilized a novel direction of clinical change analysis plan, including a group and individual analyses. Both clinicians and caregivers observed a pattern of consistent improvements in FXS-specific measures and the ABC total score, but only at the higher dose (70 mg/kg b.i.d.; *p* = 0.045 by premutation testing) (*p* = 0.045 by permutation testing) (ClinicalTrials.gov Identifier: NCT01894958). Considering similar positive results in a comparable trial of adolescents and adults with Rett syndrome, trofinetide appears to be a promising drug for neurodevelopmental disorders (ClinicalTrials.gov Identifier: NCT01703533).

Given reports of altered choline levels and cholinergic function in FXS murine models and in human studies, donepezil, an acetylcholinesterase inhibitor FDA-approved for the treatment of Alzheimer’s disease, has been initially investigated in FXS. Specifically, *FMR1* has been shown to be highly expressed in cholinergic neurons during the course of normal development [130], and choline levels were shown to be lower in persons with FXS in a small 1H magnetic resonance spectroscopy study [131]. Additionally, dysregulated cholinergic function has also been demonstrated in the subiculum of *Fmr1* KO mice [132]. In humans with FXS, a 9-subject, 6-week open-label trial of donepezil reported good drug tolerability and significant treatment-associated improvement on the ABC-C Hyperactivity and Irritability subscales [131]. Recently, results from a 12-week randomized double-blind, placebo-controlled trial of donepezil (maximum dose 5 mg per day) in 20 boys (mean age 9.1 ± 2.6 years) with full mutation FXS have been reported [133]. In this study, donepezil use was not associated with significant positive change on outcome measures employed including the Stanford-Binet Intelligence Scale, Conners 3 parent ADHD rating scale, or the Childhood Autism Rating Scale (CARS). Researchers at Stanford University recently completed a 12-week randomized double-blind, placebo-controlled, parallel-group study of donepezil (dosed 2.5 to 10 mg per day) in 42 persons with FXS (27 males, 15 females; enrolling both youth and adults under age 65) (ClinicalTrials.gov Identifier: NCT01120626), using the Contingency Naming Test (CNT) as the primary outcome measure. Analyzed results from this project are not yet available.

Sertraline, an SSRI, is known to improve BDNF levels in the CNS and to boost the deficient serotonin levels seen on positron emission tomography (PET) scanning in the brains of children with ASD who are under 5 years old [134, 135]. A randomized controlled trial of low-dose sertraline (2.5 to 5.0 mg) for 6 months in 57 young children ages 2 to 6 years with FXS was recently carried out (Greiss-Hess et al. 2016, *JDBP*, in press). Significant improvements were not shown on the primary outcome measures—the CGI-I and the expressive language subtest on the Mullen Scales of Early Learning (MSEL). However, subjects did demonstrate significant improvement on the Visual Perception subscale, Fine Motor subscale, and composite T score of the MSEL in secondary analyses. In addition, in a post-hoc analysis those children with comorbid FXS and ASD (60% of sample) demonstrated significant improvement on the Expressive Language subscale of the MSEL.

## Discussion

Despite a large number of positive preclinical drug studies in animal models of FXS, to date no approved FXS-specific drug treatments have been developed. Therefore, the treatment of FXS (mainly behavioral abnormalities) continues to be symptomatic. The numerous trial failures in the last decade could be attributed to a variety of factors; prominently trial design and outcome measures. Nevertheless, several promising areas of translational treatment and strategies to develop such treatments in FXS remain. Success in this field will be predicated in part by learning from past challenges [136, 137].

During the recent phase of significant translational research development in FXS, early failures may have been in part due to attempts to pattern FXS clinical trials after work on the FDA-approved atypical antipsychotics, aripiprazole and risperidone, for targeting irritability (aggression, self-injury, and severe tantrums) in youth with ASD. This approach was likely influenced by advice from regulatory bodies such as the FDA and others in industry, whose experience with neurodevelopmental disability drug approvals was limited and anchored specifically on the use of the ABC-C to measure irritability in registration trials. Earlier on during the development of arbaclofen for FXS, it became clear that irritability may not be the most sensitive or specific metric by which to judge FXS-specific treatments in development. This early reliance on precedents in ASD irritability-focused drug development was also likely driven by the lack of FXS-specific outcome measures. As discussed in detail in another article in this issue, the FXS field has been spending considerable efforts to develop novel and better outcome measures, but to date such instruments are not in regular use. The emphasis on using the ABC-C because it was previously vetted by the FDA may have focused trials on a behavioral irritability, a potentially less marked clinical manifestation in FXS when compared to ASD. Moreover, this focus also likely led to use of the ABC-C total score (combination of all ABC-C subscales’ scores) as an outcome, which is not a recommended or empirically supported use of the measure because a subject may have a mix of worsening and improvement in various behavioral aspects evaluated by different ABC-C subscales, thus blurring interpretation of a “total” score.

The FXS clinical trial field developed quickly marked by the need for a multi-site trial infrastructure where none previously existed. Within the last decade, industry rapidly developed molecules for large-scale placebo-controlled study in FXS. The rapid rate of clinical trials developed in FXS may have contributed to some early multi-site trial challenges. Issues such as across-site rating and assessment of enrollment criteria fidelity may have presented challenges to consistent study ratings. For example, in a study of arbaclofen in idiopathic ASD, a result was reported on the Vineland-II for only subjects assessed per protocol with a description of how many subjects did have the Vineland-II administered as requested. Such a finding may point to how having a more established multi-site trial infrastructure over time may improve study fidelity. Fortunately, developments such as the Fragile X Clinical and Research Consortium by the National Fragile X Foundation and enhanced site training over time has led to establishment of a trial infrastructure prepared to generate the most accurate and consistent trial data possible.

One important concern about the progression of the translational effort in FXS is the reliance on post-hoc analyses of preliminary studies in making critical subsequent clinical trial study design decisions for larger, more pivotal studies examining efficacy. For example, in the first AFQ056 clinical trial, a post-hoc analysis showed significant improvement in just 7 subjects with complete methylation, leading to a very substantial effort to stratify by this variable in a much larger trial. In the earlier arbaclofen trial, a post-hoc analysis showed improvement in the social avoidance subscale of the ABC [102], leading to the decision to focus on social avoidance as the primary outcome in a subsequent trial [Berry-Kravis et al.; companion paper in this journal]. In the metadoxine study, which focused on ADHD symptoms as the primary target, post-hoc analyses showed significant improvement in daily living skills, which may lead to decisions on the target for future study of this compound. It may be quite rational to design future studies based on post-hoc observations, as these adjustments may lead to demonstration of the true benefits of a compound on a different clinical problem or subgroup. However, investigators must also appreciate the risks in moving a focus of research in a new direction that is based on type II error occurring when a large number of post-hoc analyses are completed yielding one or more “false positives”. In this regard, it may be useful to consider whether several independent pieces of data suggest a similar clinical benefit, whether the post-hoc results represent clinically meaningful, not just statistically significant changes, whether the changes could be more associated with side effects of the drug rather than true improvement, and whether the clinical changes make sense based on the understanding of the neurobiological and pharmacological mechanisms of the compound under investigation.

Enhanced placebo response rates have also potentially contributed to early failures in FXS-specific treatment development. In ASD, registration trials for risperidone and aripiprazole targeting irritability reported placebo response rates in the range of 12–14% [138, 139]. In FXS, we have seen a much higher rate of placebo responders—as high as 34.6% in the arbaclofen trial—that increased the necessary treatment effect for detecting statistically significant changes [76, 102]. This issue may be driven by several factors. First, there has been great anticipation among FXS stakeholders that new treatments were on the horizon and would build from positive drug treatment data in FXS animal models. Developments like the mGluR theory of FXS received significant worldwide scientific attention and coverage in the general press, thus potentially enhancing the perception that new FXS-specific treatments being studied would be met with significant and brisk success. To date, attempts at reducing placebo effect, such as single-blind run-in treatment periods used in study of AFQ056 (mavoglurant) have had limited benefit in the field. Recent efforts to use more clinician-anchored measures performed by clinician interview show promise to reduce reliance on single parent-report measures, which may be more prone to enhanced placebo effect. For example, one effort by Neuren Pharmaceuticals focused on assessing group and individual analyses for direction of change in a diverse basket of outcomes, including both parent and clinician report. This kind of analysis is also more likely to assess changes impacting any aspect of the FXS phenotype rather than just a single behavior. As the FXS field tests treatments targeting general mechanisms, it is expected that it will be important to evaluate the entire phenotype since the degree of behavioral manifestations in different domains vary across patients. In this regard, the further development and full validation of a disease-specific outcome measure, such as the Fragile X Syndrome Rating Scale (FXSRS) used in the trofinetide trial, becomes a milestone in the process of creating better instruments for intervention studies in FXS. There is increasing evidence that individual behavioral problems in FXS may appear differently by their co-occurrence with others (e.g., anxiety and ASD) [140, 141]. In this regard, the development of a comprehensive FXS behavioral phenotype measure such as the FXSRS represents an important effort in this area. Objective measures, either performance-based measures of cognition or biomarkers (discussed below) may also be used to more accurately track treatment response with much less concern about placebo response.

There have likely been shortcomings in attempts to match outcome measures in FXS clinical trials to what may be expected from short-term treatment with drugs that rescue synaptic and other cellular pathology in mouse models of FXS. There are no clear correlates between outcomes employed in FXS animal studies and outcomes employed in initial human FXS clinical trials. For example, a drug may correct protein synthesis, dendritic spine morphology, learning, and audiogenic seizure deficits in the *Fmr1* KO mouse. Then, when moved into human studies, outcomes have included parent-report checklists focused on interfering behavior, mood, anxiety, inattention, and adaptive behavior. Thus, it is unclear what type of behavioral change would be expected with a new treatment that rescues brain connectivity, protein synthesis, and/or neuronal circuit excitability among other features targeted in *Fmr1* KO animal studies. Given this, it will be important for the field to attempt to develop testing paradigms, particularly in rodent FXS models that can be recapitulated in FXS human studies as much as possible. Despite this, and considering that species differences in FMRP and other regulatory proteins (e.g., MeCP2) exist and influence brain connectivity, it is expected that only a few phenotypes and paradigms could be applied in both rodents and humans.

The FXS translational treatment field, like most other similar fields attempting to bring treatments from bench to bedside, has not developed a sense of what age range is most appropriate to enhance chances of treatment success. While efforts in the *Fmr1* KO mouse have demonstrated phenotypic improvement in adult animals, it is possible that initial negative trial results in humans utilizing drugs with marked success in animal models of FXS may be due to missing appropriate developmental windows in the human FXS condition that best respond to treatment. This thinking has led to the concept that the earlier in life treatment is initiated, the better the chance may be of success; this has led to the upcoming study of mavoglurant in toddlers with FXS. Additionally, the duration of treatment necessary to enact a significant change in humans with FXS is not known. While improvement in behavioral and other neurobiological phenomena has occurred rapidly in FXS animal models, it is unclear if change in affected humans may take significantly longer than the short-term trial efforts to date. Longer trials have been inhibited by increased expense and other logistical issues, and by a lack of available natural history data in the field, which would allow for detection of subtle yet potentially meaningful developmental changes over time. There is also a potential that an effective FXS-specific drug treatment would enact improvements in neurobiological parameters that may actually enhance learning over time as a primary readout versus quickly providing behavioral symptomatic relief. If true, it will likely be necessary to pair new treatments with structured training/learning paradigms in order to provide standard didactic methods to a brain that has enhanced capacity to learn (synaptic plasticity) under the influence of a beneficial drug. Such efforts are ongoing in the novel, recently funded trial of mavoglurant in toddlers with FXS, where drug or placebo will be paired with a structured, standardized language-learning paradigm. Finally, extrapolations from genetically homogenous inbred mice raised in controlled settings are inherently difficult to apply to humans who, despite sharing a single gene disorder, have different genetic backgrounds and environmental exposures.

As a single gene cause of developmental disability with increasingly well-understood neurobiology, FXS has been viewed as a disorder with relative homogeneity in particular in comparison to idiopathic ASD. This led to the idea that it would be easier to find consistent druggable targets in FXS whose modulation is associated with significant symptomatic improvement across a broad section of persons with the disorder compared to treatment development in behaviorally defined, etiologically diverse syndromes. Several factors have eroded the simplicity and accuracy of this approach. As trials have occurred in FXS, it has been clear that significant phenotypical differences exist in enrollees. This has likely led to several post-hoc comparison approaches looking at various subgroups, such as those with significant social withdrawal, ASD, or anxiety. Despite being caused by dysfunctional expression of a single gene, FXS presents with real heterogeneity, including such features as presence or absence of spoken language, comorbid ASD, epilepsy, significant ADHD symptoms, and, while anxiety is one of the most consistent features of the syndrome at least in males, the severity of anxiety may significantly differ between patients. This variability even within just the male *FMR1* full mutation population clearly confounds study development and outcome assessment. This is not to mention the significant variation between the presentation of FXS in males versus females and highly variable presentation within females alone given their random X chromosome inactivation patterns. Because of this variability of presentation, it is critical to find unifying features of the disorder that are readily and reproducibly measured. Thus, efforts to address this variability using quantitative biological or bio-behavioral parameters (i.e. biomarkers) through molecular blood assays, eye tracking technologies, or electrophysiology will be important to the field. These measures will in time need to be correlated with clinical measures to show relevance to daily functioning. Hopefully, these quantitative measures can define subgroups of individuals with consistent biological abnormalities correlated with some clinical measure, or alternatively prove useful in more directly tracking drug effects on functional brain systems. Such work would not only allow for potentially more effective outcome measures, but also will provide opportunity to identify at baseline certain persons with FXS who may best respond to a particular treatment. It is clear that parsing the heterogeneity of even a single gene disorder will likely be essential to future treatment development efforts.

As a protein responsible for translational regulation of hundreds of other proteins throughout the brain, FMRP has wide-reaching impact that likely cannot be constrained to a pathogenicity focused on a single neurotransmitter or other single-pharmacological approaches. The complex sequelae of deficient FMRP represent a diverse set of known and likely many still unidentified molecular systems set into cellular dysregulation. This complexity may well render single drug approaches ineffective in the disorder. It is quite possible that combined pharmacotherapy approaches targeting a number of molecular or neural systems, all in a state of dysregulation, may be necessary to render significant clinical improvement in humans with FXS. Such approaches may mirror efforts in human immunodeficiency virus (HIV) and oncology work where combined treatments may be required to address disease.

Despite the many challenges facing the FXS translational drug development field, there are many recent developments that portend potential enhancement of future success. These include recent reports from Neuren Pharmaceuticals describing a positive phase II study result with trofinetide in older cohorts (adolescents and adults) with FXS, using a novel outcome measure analysis strategy that addresses the entire phenotype. Ongoing work to quantify EEG abnormalities across humans with FXS and the *Fmr1* KO mouse are underway in an attempt to bridge the translation treatment gap, thus enhancing the ability to predict [142] and monitor treatment response across species. Given that cognitive dysfunction is ubiquitous in FXS, treatments targeting the cognitive phenotype of the disease and development or validation of cognitive outcome measures are also important new directions for research. The FXS clinical trial field has undergone significant growth and development in recent years, thus creating a solid foundation to enhance trial fidelity across many locations. Investigators are using new models such as human induced pluripotent stem cell (iPSC) technology to develop new bench treatment assays that may aid in determining fundamental neuronal deficits in human FXS-derived cells and potentially better replicate pathology germane to the human FXS condition.

## Conclusions

Overall, the significant wave of FXS translational drug treatment development in the last decade has been marked with select improvement in FXS preclinical models that has not yet been extrapolated to human trial breakthroughs. Learning from the lessons of this experience will position the field well to move forward and enhance the opportunity for future success.

## Abbreviations

ABC: Aberrant Behavior Checklist-Community
ABC-I: ABC Irritability
ABC-SA: ABC Social Avoidance
ABC-SW: Aberrant Behavior Checklist Social Withdrawal subscale
ADHD: Attention deficit hyperactivity disorder
ADHD-RS: Attention deficit hyperactivity disorder rating scale
ADHD-RS-IV: ADHD Rating Scale 4th Edition
ALS: Amyotrophic lateral sclerosis
AMPAR: α-amino-3-hydroxy-5-methyl-4-isoxazolepropionic acid glutamate receptor
APP: Amyloid precursor protein
ASD: Autism spectrum disorder
BDNF: Brain-derived neurotrophic factor
CARS: Childhood autism rating scale
CGG: Cytosine guanine guanine
CNT: Contingency Naming Test
ERK; ERK1/2: Extracellular signal-related kinase
ERPs: Event-related potentials
FDA: Food and Drug Administration
*FMR1*: Fragile X mental retardation gene
FMRP: Fragile X mental retardation protein
FXS: Fragile X syndrome
GABA: γ-Aminobutyric acid
GSK-3b: Glycogen synthase kinase-3 beta
HIV: Human immunodeficiency virus
IGF-1: Insulin-like growth factor 1
iPSC: Induced pluripotent stem cell
ITT: Intent-to-treat
KiTAP: Test of attentional performance for children
KO: Knockout
LTD: Long-term depression
LTP: Long-term potentiation
MDX: Metadoxine ER
mGluRs: Group I metabotropic glutamate receptors
MMP-9: Matrix metalloproteinase 9
MSEL: Mullen Scales of Early Learning
mTOR: Mammalian target of rapamycin
NMDA: N-methyl-D-aspartate
PET: Positron emission tomography
PPI: Prepulse inhibition
RBANS: Repeatable Battery for the Assessment of Neuropsychological Status
SRS: Social responsiveness scale
SSRIs: Selective serotonin reuptake inhibitors
TBI: Traumatic brain injury
UTR: Untranslated region
VAS: Visual analog scale
Vineland-II: Vineland adaptive behavior scale
